# Sensitivity and specificity of Norwegian optometrists’ evaluation of diabetic retinopathy in single-field retinal images – a cross-sectional experimental study

**DOI:** 10.1186/1472-6963-13-17

**Published:** 2013-01-10

**Authors:** Vibeke Sundling, Pål Gulbrandsen, Jørund Straand

**Affiliations:** 1 Institute of Optometry and Visual Science, Faculty of Health Sciences, Buskerud University College, Kongsberg, Norway; 2 Institute of Clinical Medicine, Campus Ahus, University of Oslo, Oslo, Norway; 3 HØKH, Research Centre, Akershus University Hospital, Oslo, Norway; 4 Department of General Practice, Institute of Health and Society, University of Oslo, Oslo, Norway

**Keywords:** Diabetic retinopathy, Optometrist, Sensitivity, Specificity, Retinal images, Case finding, Screening

## Abstract

**Background:**

In the working age group, diabetic retinopathy is a leading cause of visual impairment. Regular eye examinations and early treatment of retinopathy can prevent visual loss, so screening for diabetic retinopathy is cost-effective. Dilated retinal digital photography with the additional use of ophthalmoscopy is the most effective and robust method of diabetic retinopathy screening. The aim of this study was to estimate the sensitivity and specificity of diabetic retinopathy screening when performed by Norwegian optometrists.

**Methods:**

This study employed a cross-sectional experimental design. Seventy-four optometrists working in private optometric practice were asked to screen 14 single-field retinal images for possible diabetic retinopathy. The screening was undertaken using a web-based visual identification and management of ophthalmological conditions (VIMOC) examination. The images used in the VIMOC examination were selected from a population survey and had been previously examined by two independent ophthalmologists. In order to establish a “gold standard”, images were only chosen for use in the VIMOC examination if they had elicited diagnostic agreement between the two independent ophthalmologists. To reduce the possibility of falsely high specificity occurring by chance, half the presented images were of retinas that were not affected by diabetic retinopathy. Sensitivity and specificity for diabetic retinopathy was calculated with 95% confidence intervals (CIs).

**Results:**

The mean (95%CI) sensitivity for identifying eyes with any diabetic retinopathy was 67% (62% to 72%). The mean (95%CI) specificity for identifying eyes without diabetic retinopathy was 84% (80% to 89%). The mean (95%CI) sensitivity for identifying eyes with mild non-proliferative diabetic retinopathy or moderate non-proliferative diabetes was 54% (47% to 61%) and 100%, respectively. Only four optometrists (5%) met the required standard of at least 80% sensitivity and 95% specificity that has been previously set for diabetic retinopathy screening programmes.

**Conclusions:**

The evaluation of retinal images for diabetic retinopathy by Norwegian optometrists does not meet the required screening standard of at least 80% sensitivity and 95% specificity. The introduction of measures to improve this situation could have implications for both formal optometric training and continuing optometric professional education.

## Background

Approximately 90,000 to 120,000 Norwegians have known diabetes [1], among whom reported prevalence of diabetic retinopathy (DR) ranges from 11% to 28% [2-5]. In the working age group, DR is a leading cause of visual impairment [6]. Among people with diabetes, 1% to 13% develop sight-threatening diabetic retinopathy (STDR) and 0.4% to 1.3% are visually impaired because of DR [7-13]. Regular eye examinations and early treatment of retinopathy can prevent visual loss [9,14-16], so screening for DR is cost-effective [17]. Dilated retinal digital photography with the additional use of ophthalmoscopy is the most effective and robust method of DR screening [18,19]. In Norway, the national guidelines for diabetes [20] and The Norwegian College of General Practitioners [21] recommend either regular eye examinations by an ophthalmologist or the use of retinal photography. The Norwegian Association of Optometry has issued clinical guidelines for optometric practice [22] which include guidelines for the examination and management of patients with diabetes.

People with diabetes are commonly examined in optometric practice due to having refractive errors. Norwegian optometric practice may represent a low threshold setting for case-finding of DR [23]. Studies in other countries have shown that optometrists are able to detect and grade DR [24] and specially trained optometrists perform well when screening for STDR (sensitivity 73%-97% and specificity 83%-99%) [25-29]. Since 1988, the profession in Norway has developed from being populated by opticians to being an approved healthcare profession, populated by optometrists. Consequently, Norwegian optometrists are a heterogeneous group with regard to formal education [30]. In 2004, optometrists were granted the right to prescribe diagnostic ocular drugs and since 2009 they have been able to refer patients directly to an ophthalmologist, without the patient first seeing a gate-keeping general practitioner (GP). These two responsibilities warrant a high standard of performance on the part of the optometrist.

Sensitivity and specificity define the ability of a clinical test to correctly identify people with and without a specific disease. For low prevalence diseases, a high specificity is required to avoid large numbers of false positive results. The British Diabetic Association (now Diabetes UK) has set a required screening standard for DR of at least 80% sensitivity and 95% specificity [31]. The aims of the current study were to assess the sensitivity and specificity of the optometrists’ diagnosis of DR and to assess sensitivity and specificity with respect to the optometrists’ formal education. Furthermore, we wanted to investigate how the optometrists intended to follow up their cases.

## Methods

A cross-sectional experimental design was employed. The study population, from which study participants were drawn, comprised authorized optometrists in Norway (*n*≈1850). Members of the Norwegian Association of Optometry (NOF) (*n*=1028) were invited to participate by e-mail. Only those optometrists who were currently working in private practice, who had worked in private practice for the previous 6 months and who intended to continue working in private practice for the following 6 months were eligible for inclusion in the study.

Those optometrists who responded positively to our e-mail and were subsequently accepted for inclusion in the study were sent an interactive web-based visual identification and management of ophthalmological conditions (VIMOC) examination that used Question Writer 4 software. A VIMOC examination tests clinical competency using cases and/or images with accompanying multiple choice questions [32]. The examination consisted of 14 retinal images which the optometrists were to assess with respect to the presence or absence of DR, without grading severity. Additionally, they were to decide on patient management, based solely on retinal findings and making the assumption that the patient had never been examined by an ophthalmologist. No grading scales or patient management guidelines were provided and the optometrists were not given any patient information, such as visual acuity data. It was possible to move back and forth in the VIMOC exam to review the images and revise prior assessments before submitting a final response. In addition, a questionnaire was included to gather information regarding the participants’ work experience, education, preferred method of retinal examination, methods used for retinal examination in patients with diabetes, and methods available to them for retinal examination and imaging. Optometrists used their own computers with screen resolution and colour set to maximum. Screen resolution ranged from 1024×600 to 2560×1440 pixels.

**Figure 1:**
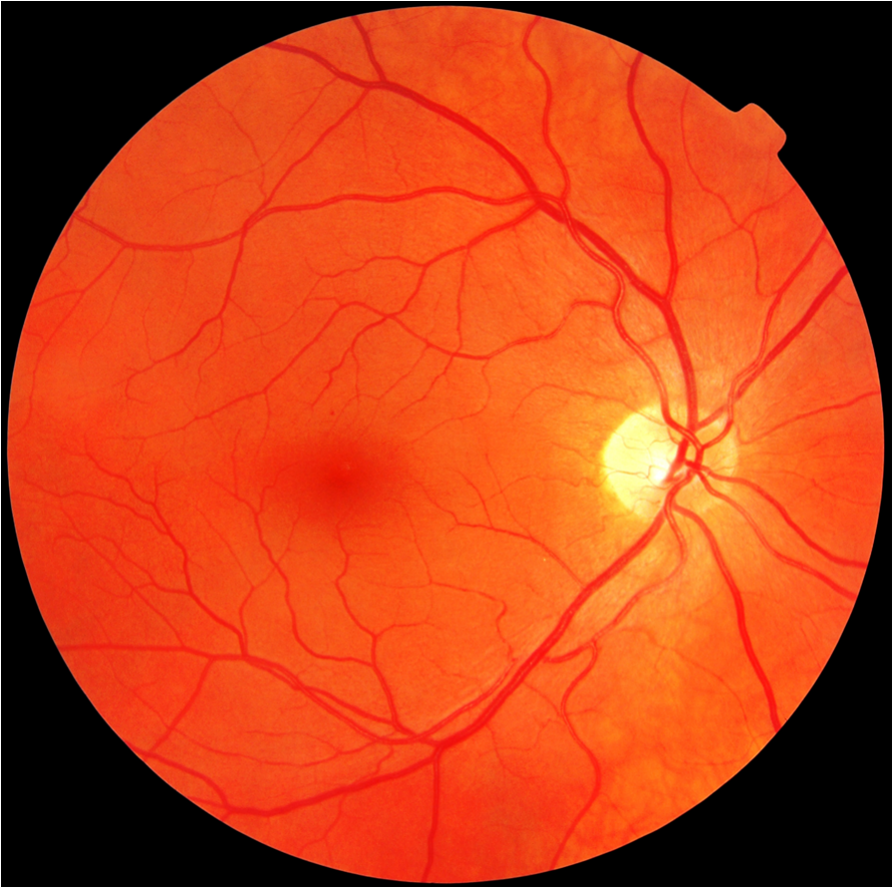
Mild non-proliferative diabetic retinopathy.

**Figure 2:**
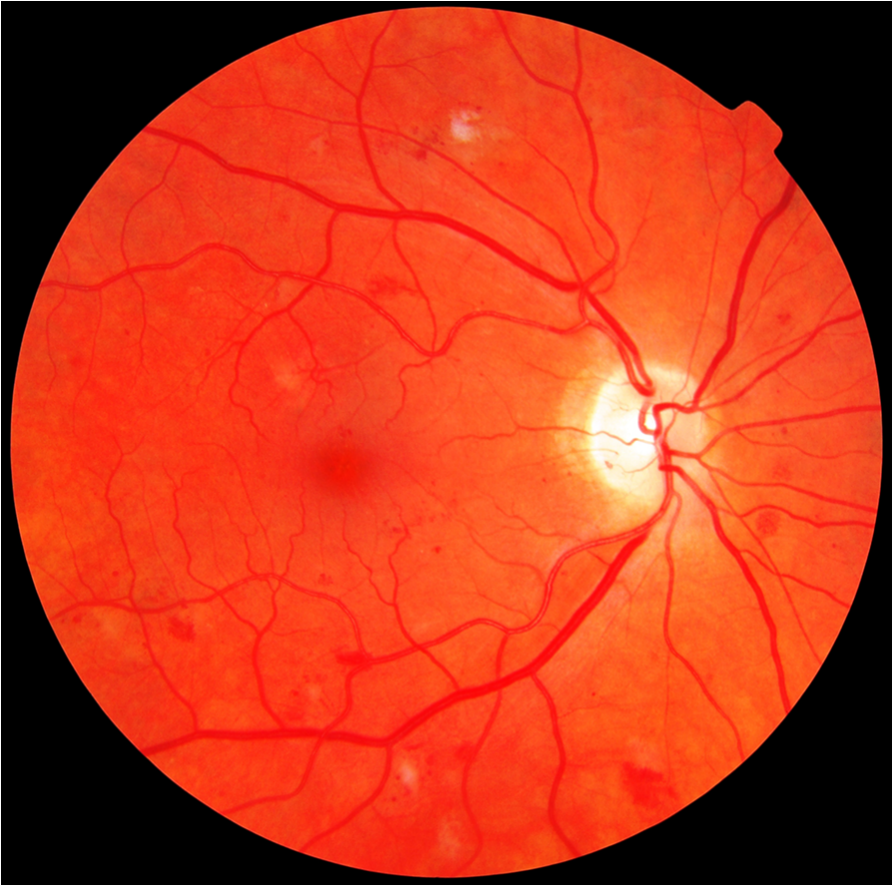
Moderate non-proliferative diabetic retinopathy.

The VIMOC retinal images were obtained from a previous Norwegian population survey [2]. The study followed the tenets of the Declaration of Helsinki for research involving humans and was approved by the Regional Committee for Medical Research Ethics, REC Central (January 19. 2009). Blinded to patient information, all images had been independently assessed by two ophthalmologists who graded the presence of retinopathy according to the Diabetic Retinopathy Disease Severity Scale [33]. The ophthalmologists viewed the images on a 21” monitor with screen resolution of 1600×1200 pixels. From a total of 239 images, only those that had been graded with full agreement between the two ophthalmologists (*n*=217) were considered for inclusion in our study. Seven images of retinas affected by non-proliferative diabetic retinopathy (NPDR) and seven images of retinas unaffected by DR were randomly selected. The DR images included five examples of mild NPDR (Figure 1) and two examples of moderate, potentially sight threatening NPDR (Figure 2). To reduce the possibility of falsely high specificity occurring by chance, half of the presented images were of retinas that were not affected by DR. The diagnoses of the two ophthalmologists for each image were used as a “gold standard” against which the performance of the study participants was assessed.

The required sample size of participants was calculated based on the following: 50% prevalence of DR in the image sample, a standard deviation of true sensitivity and specificity for individual optometrists’ image evaluation of 0.2, and 50% sensitivity and specificity for detecting DR by individual optometrists to allow maximum variance. It was calculated that a CI < 0.05 for sensitivity and specificity for any diabetic retinopathy (ADR) could be achieved with 100 study participants, meaning that sensitivity and specificity was calculated with 95% confidence interval for any retinopathy. Study participants were not asked to grade DR; the sensitivity of mild and moderate NPDR was assessed in terms of detection of retinopathy in images with mild and moderate NPDR, respectively. The screening standard established by the British Diabetic Association (Diabetes UK) of at least 80% sensitivity and 95% specificity for ADR [31] was used as the screening standard in our study. Potential associations between test performance and formal education were investigated and analysed using Pearson Chi-square and student-t tests; a p-value ≤ 0.05 was regarded as significant.

Data were collected in the period between 28th February and 14th March 2011; reminders requesting participants to complete the test were sent once.

## Results

In all, 112 (11%) members of NOF responded positively to the e-mail and volunteered to participate in this study. Of these 112, 101 (90%) met the inclusion criteria, and 74 (73%) completed the study. Participants were generally educated to a higher level than the average for Norwegian optometrists (Table 1).

**Table 1:** Characteristics of Norwegian optometrists

	**Members of NOF**^**a**^					
	**All members (*n*=1028)**		**Non-participants (*n*=954)**		**Participants (*n*=74)**	
Gender *n*, (%)						
Male	472 (46)		441 (46)		31 (42)	
Female	556 (54)		513 (54)		43 (58)	
Practice by national health region *n*, (%)						
East	338 (33)		317 (33)		21 (28)	
South	293 (29)		276 (29)		20 (27)	
West	176 (17)		165 (17)		11 (15)	
Middle	135 (13)		119 (12)		16 (22)	
North	83 (8)		77 (8)		6 (8)	
Higher education *n*, (%)						
Master of science in clinical optometry ^b,^*	200 (20)		178 (19)		22 (30)	
Private optometric practice *n*, (%) ‡	870 (88)		796 (87)		74 (100)	

Information as registered by the Norwegian Association of Optometry (NOF) and reported by the participating optometrists.

^a^ Members of the NOA February 2011.

^b^ Missing data for 37 optometrists.

Pearson Chi-square P*< 0.05 between participants and non-participants.

The optometrists’ preferred methods of retinal examination were reported to include undilated indirect ophthalmoscopy (47% of participants) and undilated retinal fundus photography (34% of participants). Multiple examination methods were reported for patients with diabetes (Table 2). Twenty-three percent of participants reported that they undertook dilated retinal examinations in patients with diabetes.

**Table 2:** Characteristics of participating optometrists by formal education

		**Master of science in clinical optometry**^**a**^	
	**All (*n*=74)**	**No (*n*=51)**	**Yes (*n*=22)**
Gender, *n* (%)			
Female	43 (58)	30 (59)	13 (59)
Male	31 (42)	21 (41)	9 (41)
Number of years as practicing optometrist, mean (sd) **	12 (±9)	10 (±8)	16 (±8)
Preferred method of retinal examination, *n* (%)			
Undilated indirect ophthalmoscopy	35 (47)	22 (43)	13 (59)
Retinal fundus photography	25 (34)	16 (31)	8 (36)
Undilated direct ophthalmoscopy	9 (12)	9 (17)	0 (0)
Other	5 (7)	4 (8)	1 (1)
Retinal examinations methods used in patients with diabetes, *n* (%)			
Undilated retinal photography	46 (62)	30 (59)	15 (68)
Undilated indirect ophthalmoscopy*	39 (53)	23 (45)	16 (73)
Dilated indirect ophthalmoscopy	15 (20)	9 (18)	6 (27)
Dilated retinal photography	11 (15)	8 (16)	3 (14)
Undilated direct ophthalmoscopy*	11 (15)	11 (22)	0 (0)
Available instruments for retinal examination and imaging, *n* (%)			
Direct ophthalmoscope and/or indirect slit-lamp ophthalmoscopy	71 (96)	48 (94)	22 (100)
Retinal fundus camera	65 (88)	44 (86)	20 (91)
Scanning-laser ophthalmoscope (Optomap)	19 (26)	10 (20)	9 (41)

^a^ Missing data for 1 participant.

Student t-test P*<0.05 and P**<0.01 between optometrists with and without MSc in clinical optometry.

The optometrists’ assessments of each of the 14 VIMOC images are presented in Table 3. Optometrists with higher optometric education (a Master of Science in clinical optometry [MSc]) demonstrated significantly higher sensitivity than those who had a basic optometric education (Table 4). The specificity was not influenced by the optometrist education level.

**Table 3:** Optometrists’ VIMOC evaluations of retinal images and corresponding ophthalmologist grading and patient glucose status

		**Image evaluation**			**Optometrist consideration on further management**					
**Image**^**a**^	**Patient status**	**Ophthalmologists grading of DR**	**Optometrists true findings of DR**		**No/Routine follow-up**		**Report / referral to general practitioner**		**Report / referral to ophthalmologist**	
			***n***	**% (95%CI)**	***n***	**% (95%CI)**	***n***	**% (95%CI)**	***n***	**% (95%CI)**
8	IGT	No DR	46	64 (52 to 75)	17	37 (23 to 51)	21	46 (31 to 60)	8	17 (6 to 28)
5	KDM	No DR	53	72 (61 to 82)	30	37 (43 to 70)	10	19 (8 to 29)	13	25 (13 to 36)
14	SDDM	No DR	61	82 (74 to 91)	31	51 (38 to 63)	18	30 (18 to 41)	12	20 (10 to 30)
13	SDDM	No DR	64	86 (79 to 94)	33	52 (39 to 64)	10	16 (7 to 25)	21	33 (21 to 44)
12	SDDM	No DR	68	92 (86 to 98)	58	85 (77 to 94)	4	6 (0 to 11)	6	9 (2 to 16)
11	KDM	No DR	71	96 (91 to 100)	61	86 (78 to 94)	2	3 (0 to 7)	8	11 (4 to 19)
2	NGT	No DR	73	99 (96 to 100)	66	90 (84 to 97)	4	5 (0 to 11)	3	4 (0 to 9)
1	NGT	Mild NPDR	23	31 (20 to 42)	1	4 (0 to 13)	8	35 (15 to 54)	14	61 (41 to 81)
6	IGT	Mild NPDR	39	53 (41 to 64))	1	3 (0 to 8)	10	26 (12 to 39)	28	72 (58 to 86)
3	NGT	Mild NPDR	40	54 (42 to 66)	0	0 (0 to 0)	19	48 (32 to 63)	21	53 (37 to 68)
7	DM	Mild NPDR	41	55 (44 to 67)	1	2 (0 to 7)	15	37 (22 to 51)	25	61 (46 to 76)
4	DM	Mild NPDR	57	77 (67 to 87)	2	4 (0 to 8)	20	35 (23 to 47)	35	61 (49 to 74)
9^b^	DM	Moderate NPDR	71	100 (100 to 100)	0	0 (0 to 0)	4	6 (0 to 11)	67	94 (89 to100)
10^c^	DM	Moderate NPDR	73	100 (100 to 100)	0	0 (0 to 0)	8	11 (4 to 18)	65	89 (82 to 96)

CI, confidence interval; DR, diabetic retinopathy; IGT, impaired glucose tolerance; KDM, known diabetes; NGT, normal glucose tolerance; NPDR, non-proliferative diabetic retinopathy; SDDM, screen-detected diabetes; VIMOC, Visual Identification and Management of Ophthalmological Conditions.^ a^ The number refers to the number in image presentation sequence of the VIMOC examinationMissing grading data from ^b^3 and ^c^1 optometrists.

**Table 4:** Optometrists’ sensitivity and specificity for identifying diabetic retinopathy, presented by formal education level

	**Sensitivity**			**Specificity**
	**Any DR (*n *=7)**	**Mild DR (*n *=5)**	**Moderate DR (*n *=2)**	**No DR (*n *=7)**
	**% (95%CI)**	**% (95%CI)**	**% (95%CI)**	**% (95%CI)**
All optometrists (*n*=74)	67 (62 to 72)	54 (47 to 61)	100	84 (80 to 89)
Formal education ^a,**^				
BSc or lower (*n*=51)	63 (56 to 69)	48 (39 to 57)	100	84 (78 to 89)
MSc (*n*= 22)	77 (71 to 84)	68 (59 to 77)	100	85 (77 to 93)

BSc, Bachelor of science; CI, confidence interval; DR, diabetic retinopathy; MSc, master of science.

^a^ Missing data for 1 optometrist.

Student t-test P**<0.01 statistically significant difference in sensitivity between optometrists with MSc and optometrists with BSc or even lower formal education.

No association was found either between sensitivity or specificity and the number of years of experience in optometric practice, or between sensitivity or specificity and the participants’ preferred method of retinal examination. The screening standard for sensitivity of at least 80% and specificity of at least 95%, for ADR, was met by 24 (32%) and 31 (42%) optometrists, respectively, overall. The standards for sensitivity and specificity were met by 50% and 45%, respectively, of optometrists who held an MSc and by 25% and 39%, respectively, who had a basic optometric education. Only four optometrists (5%) met the required standard for both sensitivity and specificity.

Patient management decisions were dependent on retinal findings (Table 5). Report/referral to a GP and/or an ophthalmologist was regarded as appropriate for 99% and 96% of true- and false-positive findings, respectively. The rate of referral to an ophthalmologist was higher for moderate than for mild NPDR (92% vs. 62%). No further management was considered appropriate in 68% and 66% of cases of true- and false-negative findings, respectively.

**Table 5:** Individual image evaluation and suggested follow-up

	**Images with diabetic retinopathy (*n *=518)**				**Images without diabetic retinopathy (*n *=518)**			
	**True positive sensitivity**		**False negative**		**True negative specificity**		**False positive**	
	*n*	%	*n*	%	*n*	%	*n*	%
Screening standard set to meet ^a^	414	80	104	20	492	95	26	5
	*n*	% (95%CI)	*n*	% (95%CI)	*n*	% (95%CI)	*n*	% (95%CI)
Optometrists’ image evaluation	348	67 (62 to 72)	170	32 (28 to 38)	437	84 (80 to 89)	81	16 (11 to 20)
Further management^b^								
None / Routine follow up	5	1 (0 to 3)	113	66 (59 to 74)	296	68 (64 to 72)	3	4 (0 to 8)
Report / referral to general practitioner	84	24 (20 to 29)	41	24 (18 to 21)	69	16 (12 to 19)	39	48 (37 to 59)
Report / referral to ophthalmologist	255	74 (70 to 79)	16	9 (5 to 14)	71	16 (13 to 20)	39	48 (37 to 59)

^a^ British Diabetic Association. Retinal photographic screening for diabetic eye disease. A British Diabetic Association Report. London: British Diabetic Association; 1997.

^b^ Data missing for 5 image evaluations.

## Discussion

Only 5% of the responding optometrists satisfied the screening standard established by the British Diabetic Association of at least 80% sensitivity and 95% specificity [31]. Overall, sensitivity for detecting ADR was low and specificity was moderate. Sensitivity for detecting potential STDR was, however, high. This suggests that the optometrists’ assessment of retinal images is an unreliable method of screening for DR. The sensitivity and specificity of detection of DR in the current study and previous studies is presented in Table 6. It is not possible to make a direct comparison between the current study and previous studies that involved community optometrists [28,34], as those studies did not report sensitivity and specificity levels for individual optometrists. However, based on the reported mean levels of sensitivity and specificity, it is unlikely that individual optometrists in those studies would have met the British Diabetic Association screening criteria. The sensitivity for detecting ADR in our study (67%) was lower than that reported by Gibbins et al. (86-88%) [28]. However, the sensitivity for detecting *STDR* was higher in our study than in either the Gibbins et al. or Buxton et al studies (100% vs. 47-97%) [28,34] and the specificity was similar (84% vs. 83-95%). The greater sensitivity for detecting STDR in the current study could have been a result of the higher prevalence of STDR in our VIMOC sample compared with these earlier studies, which may have inflated sensitivity by chance. The prevalence of ADR in our study was comparable with the prevalence of ADR in the study by Gibbins et al. [28]. In that study, optometrists had received special training in the identification and grading of DR, which could explain the relatively high sensitivity levels observed.

**Table 6:** Optometrists’ sensitivity and specificity for identifying diabetic retinopathy as reported in the current study and previous studies

		**Sensitivity (95%CI)**		**Specificity (95%CIn**	
**Study**	**Retinal examination method**	**ADR**	**STDR**	**ADR**	**STDR**
Our study (2011)					
Community optometrists	Image evaluation of digital images	67 (62 to 72)		84 (80 to 89)	
Harvey et al (2006)					
Optometrists in a screening program	Not available		80 (71 to 89)		99 (98 to 100)
Olson et al (2003)					
Specially trained optometrists	Dilated slit-lamp examination		73 (52 to 88)		90 (87 to 93)
Schmid et al (2002)					
Community optometrists	Ophthalmoscopy (free choice)	92 (84 to 100)		94 (90 to 98)	
	Image evaluation of retinal slides	94 (90 to 98)		97 (92 to 100)	
Hulme et al (2001)					
Specially trained optometrists	Dilated slit-lamp examination	72	87	77	91
Prasad et al (2001)					
Specially trained optometrists	Dilated slit-lamp examination	66 (65 to 67)	76 (70 to 81)	97 (97 to 98)	95 (95 to 96)
Gibbins et al (1998)					
Community optometrists	Image evaluation of 35 mm slides	88 (83 to 93)	91 (79 to 98)	68 (58 to 68)	83 (79 to 87)
Specially trained optometrist	Image evaluation of 35 mm slides	86 (81 to 91)	97 (90 to 100)	89 (85 to 93)	87 (84 to 91)
Buxton et al (1991)					
Community optometrists	Image evaluation of Polaroid images	48 (26 to 69)		94 (92 to 97)	

ADR, any diabetic retinopathy, STDR, sight-threatening diabetic retinopathy.

We have found that sensitivity, but not specificity, was influenced by the level of formal education the participants had received. Optometrists with an MSc had a significantly higher sensitivity than optometrist with a basic optometric education. This suggests that our results give a better estimate of sensitivity and specificity in general optometric practice, as our study included optometrists who had not had any special training in screening for DR.

The sensitivity observed in this study is in line with that observed in a previous study we undertook to investigate Norwegian general optometric practice [23], where sensitivity ranged from 61% to 65%, based on an assumption of 14% prevalence of DR among patients with diabetes [3]. Specificity in the current study was, however, lower than in our previous study (84% vs. 98-100%), which could be explained by the difference in prevalence between the two studies. In the current study, 98% of findings of DR (both true- and false-positives) were considered to warrant a report/referral to a physician. This is higher than the rate of 57% reported in a national practice registration in Norway [23]. The experimental design in our study, where optometrists were blinded to patient information but assumed that the patient had never been examined by an ophthalmologist, may have led to an increased tendency to recommend referral to a physician.

Assuming the prevalence of DR is 14% [3], the negative and positive predictive values of the optometrists’ evaluation of DR in our study would be 94% and 41%, respectively. Based on this and on the fact that Norwegian optometrists undertake approximately 1 million eye examinations per year (of which approximately 4% are in patients with diabetes [30]), our findings suggest that each year approximately 5500 patients without DR are referred based on a false positive result, while in approximately 1300 patients with DR, no further action is taken. However, if the British Diabetic Association screening criteria were met in Norwegian optometric practice, these figures would be 1700 and 800, respectively. Our results suggest that an excessive workload is being placed on healthcare services by inaccurate referral practices. However, the national guidelines recommend eye examination by ophthalmologists [20,21], thus the report/referral of a patient who has not previously been seen by an ophthalmologist should not be discouraged. Of greater concern is the false security given to those patients with DR who are not referred to an ophthalmologist.

The strengths of this study are the use of standardised images in the VIMOC exam and the use of a diagnostic “gold standard” based on 100% agreement between two independent ophthalmologists. The experimental design allowed the calculation of sensitivity and specificity with acceptable precision in a relatively large nationwide sample of optometrists, something that was not achieved in previous studies [25-28,34]. In terms of gender, number of years in practice and geographical location, our sample of optometrists is representative of members of the NOF and of optometrists who participated in a previous study of Norwegian optometric practice [23,30]. The potential for knowledge bias and overestimation of sensitivity and specificity for general optometric practice was reduced in the current study because the optometrists were not provided with grading scales, nor were they given specific training prior to the study.

One potential limitation of the study was the possibility of selection bias, as optometrists with a specific interest in diabetes may have been more likely to accept the invitation to participate and hence may have been overrepresented in the study. This could have inflated the sensitivity levels observed, compared with general optometric practice. On the other hand, participating optometrists did not have specific training in screening for DR, nor were they provided with a DR grading scale or a computer screen that would facilitate classification of DR. Variable viewing conditions may have influenced the detection rate of DR. Small screen size, low screen resolution and inadequate colour setting may have led to lower sensitivity for detecting mild DR. On the other hand, the optometrists’ use of their own facilities simulated real practice, something that the use of perfect viewing conditions could not have done.

## Conclusions

Our study is likely to have given a better representation of general optometric practice than previous studies [25-28,34]. However, our findings indicate that at present case-finding of DR in Norwegian optometric practice is unreliable. Formal optometric training in screening for DR and continuing education may improve diagnostic sensitivity. Further research will be needed to evaluate the effectiveness of measures undertaken to improve optometrists’ diagnostic accuracy for case-finding of DR.

## Abbreviations

ADR: Any diabetic retinopathy; CI: Confidence interval; DR: Diabetic retinopathy; GP: General Practitioner; MSc: Master of Science in clinical optometry; NOF: Norwegian Association of Optometry; NPDR: Non-proliferative diabetic retinopathy; STDR: Sight-threatening diabetic retinopathy; VIMOC: Visual identification and management of ophthalmological conditions.

## Competing interests

The authors declare that they have no financial or non-financial competing interests.

## Authors’ contributions

VS conceived of the study and participated in its design, acquired and statistically analysed the data and drafted the manuscript. PG and JS participated in the design of the study and critically revised the manuscript. All authors read and approved the final manuscript.

## Authors’ information

VS is the program coordinator for postgraduate courses in Optometry and Visual Science at the Department of Optometry and Visual Science, Faculty of Health Sciences, Buskerud University College.

## Pre-publication history

The pre-publication history for this paper can be accessed here:

http://www.biomedcentral.com/1472-6963/13/17/prepub

## References

[B1] Stene LC Midthjell K Jenum AK Skeie S Birkeland KI Lund E Joner G Tell GS Schirmer H Hvor mange har diabetes mellitus i Norge?. [Prevalence of diabetes mellitus in Norway] Tidsskr Nor Laegeforen 2004 124 11 1511 1514 15195154

[B2] Sundling V Platou CG Jansson RW Bertelsen G Wøllo E Gulbrandsen P Retinopathy and visual impairment in diabetes, impaired glucose tolerance and normal glucose tolerance: the Nord-Trondelag Health Study (the HUNT study) Acta Ophthalmol 2012 90 3 237 243 10.1111/j.1755-3768.2010.01998.x 20809910

[B3] Hapnes R Bergrem H Diabetic eye complications in a medium sized municipality in southwest Norway Acta Ophthalmol Scand 1996 74 5 497 500 8950402 10.1111/j.1600-0420.1996.tb00607.x

[B4] Kilstad HN Sjolie AK Goransson L Hapnes R Henschien HJ Alsbirk KE Fossen K Bertelsen G Holstad G Bergrem H Prevalence of diabetic retinopathy in Norway: report from a screening study Acta Ophthalmol 2012 90 7 609 612 10.1111/j.1755-3768.2011.02160.x 21955522

[B5] Bertelsen G Peto T Lindekleiv H Schirmer H Solbu MD Toft I Sjolie AK Njolstad I Tromso eye study: prevalence and risk factors of diabetic retinopathy Acta Ophthalmol 2012 10.1111/j.1755-3768.2012.02542.x. [Epub ahead of print] 22994366

[B6] Porta M Bandello F Diabetic retinopathy A clinical update Diabetologia 2002 45 12 1617 1634 10.1007/s00125-002-0990-7 12488951

[B7] Olafsdottir E Andersson DK Stefansson E Visual acuity in a population with regular screening for type 2 diabetes mellitus and eye disease Acta Ophthalmol Scand 2007 85 1 40 45 17244208 10.1111/j.1600-0420.2006.00753.x

[B8] Scanlon PH Foy C Chen FK Visual acuity measurement and ocular co-morbidity in diabetic retinopathy screening Br J Ophthalmol 2008 92 6 775 778 10.1136/bjo.2007.128561 18356262

[B9] Zoega GM Gunnarsdottir T Bjornsdottir S Hreietharsson AB Viggosson G Stefansson E Screening compliance and visual outcome in diabetes Acta Ophthalmol Scand 2005 83 6 687 690 10.1111/j.1600-0420.2005.00541.x 16396645

[B10] Jeppesen P Bek T The occurrence and causes of registered blindness in diabetes patients in Arhus County, Denmark Acta Ophthalmol Scand 2004 82 5 526 530 10.1111/j.1600-0420.2004.00313.x 15453847

[B11] Prasad S Kamath GG Jones K Clearkin LG Phillips RP Prevalence of blindness and visual impairment in a population of people with diabetes Eye 2001 15 Pt 5 640 643 11702977 10.1038/eye.2001.200

[B12] Broadbent DM Scott JA Vora JP Harding SP Prevalence of diabetic eye disease in an inner city population: the Liverpool Diabetic Eye Study Eye 1999 13 160 165 10.1038/eye.1999.43 10450374

[B13] Hove MN Kristensen JK Lauritzen T Bek T The prevalence of retinopathy in an unselected population of type 2 diabetes patients from Arhus County, Denmark Acta Ophthalmol Scand 2004 82 4 443 448 10.1111/j.1600-0420.2004.00270.x 15291939

[B14] Backlund LB Algvere PV Rosenqvist U New blindness in diabetes reduced by more than one-third in Stockholm County Diabet Med 1997 14 9 732 740 10.1002/(SICI)1096-9136(199709)14:9<732::AID-DIA474>3.0.CO;2-J 9300222

[B15] Kristinsson JK Hauksdottir H Stefansson E Jonasson F Gislason I Active prevention in diabetic eye disease. A 4-year follow-up Acta Ophthalmol Scand 1997 75 3 249 254 9253967 10.1111/j.1600-0420.1997.tb00766.x

[B16] Stefansson E Bek T Porta M Larsen N Kristinsson JK Agardh E Screening and prevention of diabetic blindness Acta Ophthalmol Scand 2000 78 4 374 385 10.1034/j.1600-0420.2000.078004374.x 10990036

[B17] Javitt JC Aiello LP Cost-effectiveness of detecting and treating diabetic retinopathy Ann Intern Med 1996 124 1 Pt 2 164 169 8554212 10.7326/0003-4819-124-1_part_2-199601011-00017

[B18] Hutchinson A McIntosh A Peters J O’Keeffe C Khunti K Baker R Booth A Effectiveness of screening and monitoring tests for diabetic retinopathy–a systematic review Diabet Med 2000 17 7 495 506 10.1046/j.1464-5491.2000.00250.x 10972578

[B19] Gillibrand W Broadbent D Harding S Vora J The English national risk-reduction programme for preservation of sight in diabetes Mol Cell Biochem 2004 261 1–2 183 185 15362502 10.1023/b:mcbi.0000028754.70862.13

[B20] Helsedirektoratet Nasjonale faglige retningslinjer (IS-1674). Diabetes - Forebygging, diagnostikk og behandling [National professional guidelines. Diabetes - Prevention, diagnostics and treatment] 2009 Oslo: Helsedirektoratet

[B21] Claudi T Cooper JG Midthjell K Daae C Furuseth K Hanssen KF NSAMs handlingsprogram for diabetes i allmennpraksis 2005 (NSAMs guidelines for diabetes in general practice 2005) 2005 7652730

[B22] Optikerforbund N Retningslinjer i klinisk optometri 2005 Oslo: Norges Optikerforbund

[B23] Sundling V Gulbrandsen P Bragadottir R Bakketeig LS Jervell J Straand J Suspected retinopathies in Norwegian optometric practice with emphasis on patients with diabetes: a cross-sectional study BMC Health Serv Res 2008 8 38 10.1186/1472-6963-8-38 18261204 PMC2262885

[B24] Schmid KL Swann PG Pedersen C Schmid LM The detection of diabetic retinopathy by Australian optometrists Clin Exp Optom 2002 85 4 221 228 10.1111/j.1444-0938.2002.tb03041.x 12135414

[B25] Olson JA Strachan FM Hipwell JH Goatman KA McHardy KC Forrester JV Sharp PF A comparative evaluation of digital imaging, retinal photography and optometrist examination in screening for diabetic retinopathy Diabet Med 2003 20 7 528 534 10.1046/j.1464-5491.2003.00969.x 12823232

[B26] Hulme SA Tin UA Hardy KJ Joyce PW Evaluation of a district-wide screening programme for diabetic retinopathy utilizing trained optometrists using slit-lamp and Volk lenses Diabet Med 2002 19 9 741 745 10.1046/j.1464-5491.2002.00677.x 12207810

[B27] Prasad S Kamath GG Jones K Clearkin LG Phillips RP Effectiveness of optometrist screening for diabetic retinopathy using slit-lamp biomicroscopy Eye 2001 15 Pt 5 595 601 11702969 10.1038/eye.2001.192

[B28] Gibbins RL Owens DR Allen JC Eastman L Practical application of the European Field Guide in screening for diabetic retinopathy by using ophthalmoscopy and 35 mm retinal slides Diabetologia 1998 41 1 59 64 10.1007/s001250050867 9498631

[B29] Harvey JN Craney L Nagendran S Ng CS Towards comprehensive population-based screening for diabetic retinopathy: operation of the North Wales diabetic retinopathy screening programme using a central patient register and various screening methods J Med Screen 2006 13 2 87 92 10.1258/096914106777589669 16792832

[B30] Sundling V Gulbrandsen P Bragadottir R Bakketeig LS Jervell J Straand J Optometric practice in Norway: a cross-sectional nationwide study Acta Ophthalmol Scand 2007 85 6 671 676 10.1111/j.1600-0420.2007.00929.x 17408386

[B31] British Diabetic Association Retinal photographic screening for diabetic eye disease. A British Diabetic Association Report 1997 London: British Diabetic Association

[B32] Aakre BM Svarverud E Utdanning for klinisk kompetanse (Education towards clinical competence) Optikeren 2011 5 52 53

[B33] Wilkinson CP Ferris FL 3rd Klein RE Lee PP Agardh CD Davis M Dills D Kampik A Pararajasegaram R Verdaguer JT Proposed international clinical diabetic retinopathy and diabetic macular edema disease severity scales Ophthalmology 2003 110 9 1677 1682 10.1016/S0161-6420(03)00475-5 13129861

[B34] Buxton MJ Sculpher MJ Ferguson BA Humphreys JE Altman JF Spiegelhalter DJ Kirby AJ Jacob JS Bacon H Dudbridge SB Screening for treatable diabetic retinopathy: a comparison of different methods Diabet Med 1991 8 4 371 377 10.1111/j.1464-5491.1991.tb01612.x 1830260

